# Additionality and Charitable Support for Public Services: Community Fundraiser Perspectives in a Tax-Funded National Health Service

**DOI:** 10.1332/20408056Y2026D000000095

**Published:** 2026-08-31

**Authors:** Steph Haydon, Ellen Stewart, Francesca Vaghi

**Affiliations:** University of Kent, United Kingdom; University of Glasgow, United Kingdom; Western Norway University of Applied Sciences, Norway

## Abstract

Additionality refers to the idea that charitable contributions to publicly-funded services should enhance, not replace, state provision. Whilst it is often assumed that donors will avoid substitution of public funding when seeking causes to support, longstanding traditions of charitable support for public services (e.g. schools, healthcare facilities) endure. Through four case studies of charitable activities supporting NHS hospitals in the UK, we examine the perspectives of community fundraisers and the staff who work with them. Professional fundraisers often described a tension between selling their cause while conveying its additionality. Meanwhile, many community fundraisers were initially drawn to support healthcare services to address identified shortfalls in provision. Once involved in fundraising efforts, participants often develop a deeper commitment to the social experience of taking part. This generates ongoing engagement which largely overrides additionality concerns, and which limits the potential for fundraising to be redirected to alternative causes.

## 1 Introduction

Additionality is the principle which governs the role of charitable contributions to predominantly state-funded public services. It refers to the idea that charitable contributions should enhance, rather than replace, core provisions, providing nice-to-have, but inessential, extras (Schuster, 1994). In theory, this should safeguard the beneficial aspects of charitable contributions – including positive community engagement and a degree of public voice in services (for example Paine et al, 2019) – whilst protecting equitable access between areas which attract charitable moneys and those which do not. Existing research has explored charitable provision of a wide range of (formerly) public services (Rees and Mullins, 2016), including libraries (Forkert, 2016), leisure centres (Nichols et al, 2020) and schools (Wilde et al, 2016), often arguing that charity is plugging gaps left by state retreat during austerity. This literature has particularly explored the outsourcing of entire services to charitable control. We focus instead on additionality in a context where charity contributes to, or supplements the work of statutory public services. This paper examines the motivations and experiences of community fundraisers in the UK’s National Health Service (NHS), focusing on how they perceive and navigate issues of additionality in their fundraising practice.

Broader debates on whether the presence of state funding crowds out (by disincentivising) or crowds in (by fostering egalitarian views) private contributions are longstanding (Müller & Rau, 2020). Breeze & Mohan (2016) report attitudinal data on the respective roles of charity and the state, including modest increases in the proportion of UK survey respondents who see selected areas of expenditure as the responsibility of charity between 1991 and 2015. This is true across the illustrative categories of expenditure, although the example of ‘kidney machines’ indicates more resilient commitment to state responsibility, and respite holidays for the disabled are considered in the “nice but not necessary” category of charitable responsibilities.

This population-level attitudinal data, though, should be distinguished from the attitudes of donors and fundraisers. Across the literature, and across different causes, the possibility that funds raised will replace, rather than supplement, statutory provision is broadly assumed to dissuade donors from giving (Breeze, 2010; Garg et al, 2012). In reality, research tends to find that the impact on donor choices will be heterogenous, depending on individual and group motivations (Cheslock and Gianneschi, 2008). Researchers also acknowledge that care for a particular cause can supersede concerns about additionality (Breeze, 2010). However, research on charitable action often focuses on major donors, and neglects the perceptions and experiences of community fundraisers; people who choose to give up their time and money to volunteer and fundraise (Rider, 2019; Chapman et al, 2022).

Charitable contributions to healthcare in the UK is a particularly intriguing field in which to explore additionality because of both its policy history (Harris and Cresswell, 2023), and a general lack of public awareness of the continued extent of charitable contributions to healthcare (Abnett et al, 2023; Stewart and Dodworth, 2023). It is a particularly unusual case from the point of view of the USA, where ‘grateful patient’ donations to healthcare are normalised (Collins et al, 2018; Jagsi et al, 2020). In the UK, a founding principle of the NHS was that responsibility for funding and delivering hospital services should lie with the state. Minister of Health, and architect of the NHS, Aneurin Bevan famously argued that reliance on private charity for hospital funding was “repugnant to a civilised community” (HC Deb, 1946). Rather than exclude all charitable contributions, it was envisaged that charity would shift from providing essential services to supplementing them with ‘extras’: “a theoretical border between state-funded essentials and charitably financed inessentials was drawn” (Blythe and Gorsky, 2026). Whilst some early commentators provided examples of what they thought such ‘extras’ might include – primarily funding research, and providing staff and patient amenities (British Hospitals Association, 1948) – no policy has ever clearly demarcated the essential-extra binary in the NHS (Abnett et al, 2023). Today, the distinction remains blurry and difficult to operationalise.

Given the persistence of community fundraising for hospitals in the UK and the continued lack of attention on community fundraising practice, we explore perspectives on additionality among community fundraisers for hospitals in the NHS. In the NHS, fundraising initiatives are carried out both by amateur Leagues of Friends (Paine et al, 2019; Millward, 2023) and by professionalised NHS charities (Abnett et al, 2023), with community fundraisers present in both types of organisation. Through four case studies of charitable activities supporting NHS hospitals, we explore how community fundraisers practice additionality, moving beyond accounts of it as either an ethical principle or a legal criteria. Our paper demonstrates how committed community fundraisers navigate additionality, mobilising it strategically in particular moments, but then backgrounding it as a nice, but currently unrealistic aspiration, when concerns for the quality of local provision arise.

## 2 Background: community fundraising for public services

When examining local fundraising for publicly-funded services, useful insights can be found in research on fundraising for schools. Body (2017, 2020) has documented local fundraising for schools in England, where activities from bake sales to school fairs have long been the norm. Here, fundraising income is typically used to fund activities viewed as inessential, such as school trips and equipment, whilst excluding teachers’ salaries (deemed ‘essential’): “Although the case study schools highlighted deficits in their budget as putting pressure on the school, they were clear to focus fundraising asks on going beyond what state funding should cover” (Body, 2017:3–4). This points to normative views held by school teachers about the state’s responsibility, as well as their assumptions of donor preferences to not substitute for state provisions. However, several years of public funding cuts later, Body (2020:99) observed that “schools are becoming reliant on voluntary action to deliver core statutory services”, such as by replacing support staff with volunteers. Thus, in contexts of reduced public funding, where schools are faced with the decision of providing a service through voluntary initiative or not providing it at all, many turn to charity for support.

Much literature on fundraising for publicly-funded services is preoccupied by the impact on inequalities between communities. In education, fundraisers in more affluent areas may have access to social and cultural capital that will guarantee the success of fundraising endeavours; fundraising for schools in less affluent areas might prove less effective (Body, 2017). This can further embed educational inequalities because family and community affluence are positively correlated with student enrolment and attainment (Schnabel, 2024). One Canadian study of school fundraising inequities argued that they “constitute new structural inequities, symbolic domination and spatial violence” (Yoon et al, 2025).

Likewise, in the English NHS, there is evidence that charitable income is unevenly distributed between Trusts: Specialist Trusts and Trusts serving more affluent populations tend to receive a greater share of their income from charity (Bowles et al., 2023; Mohan & Clifford 2024). Research has also identified inequality between charitable support for Health Boards in Scotland (Stewart and Dodworth, 2023). This is especially concerning given many charities provide what could reasonably be considered essential equipment (e.g. x-ray machines) and furniture (e.g. mattresses) (Abnett et al., 2023). Charitable fundraising in publicly-funded services may therefore increase inequalities in provision: given deeply embedded disparities in community capital, larger reliance on fundraising for community services risks an overt ‘postcode lottery’ of those aspects deemed essential to provision.

Existing research on charitable monies in the NHS variously depicts additionality as either a legal principle, or merely an informal convention (see for example Paines, 2007). In a witness seminar on hospital fundraising (Gorsky and Arnold-Forster, 2024), participants suggested that there had been a shift over time. One prominent fundraiser from the 1980s onwards declared:

“Throughout the nineties there was still that dividing line between what’s the Government’s responsibility and what was seen to be additional and charitable” (Marion Allford, quoted in Gorsky and Arnold-Forster, 2024).

Ellie Orton, Chief Executive of the umbrella membership organisation for local NHS Charities argued for a shift towards more impactful, considered role, dismissing earlier efforts to claim the additionality of purchased items:

“it needs to be done strategically, so that it can have most impact, rather than oh, yeah it’s a piece of equipment that’s got a couple more buttons – so we can call it ‘additional’ and so the Charity can pay for it.”

These quotes both suggest a perception of changing practice over time, with a ‘dividing line’ still guiding one participant’s practice in the 1990s, but a contrasting desire from Orton to define the role of NHS charities as being *more* significant, rather than bracketed into ‘extra’ items.

## 3 Literature on fundraising motivations

A central pillar in debates on additionality are motivations: why donors and fundraisers engage in charitable behaviour will shape what they are and are not willing to contribute to, including whether they are willing to support essential services and the influence of state contributions on their charitable decisions. In this literature review, we explore different perspectives on donor motivations and the pivotal role of fundraisers in shaping charitable decisions.

### 3.1 Motivations for charitable behaviour as socio-relational

With ‘charitable behaviour’ we refer to contributions of both time (i.e. volunteering) and money (i.e. donating) for public benefit. The motivations driving such behaviour are generally classed as altruistic or self-interested (Adloff, 2015), and intrinsic (e.g. psychological benefits) or extrinsic (e.g. reputational gains) (Pharoah, 2016). Moving beyond such individualistic conceptualisations, Schervish and Havens (2002) posit that charitable behaviour is driven by identification with the proposed beneficiary: identifying with the needs of others generates empathy and a caring response, which can take the form of charitable behaviour. Resulting interactions between donors/volunteers and beneficiaries are reciprocal as both parties give to and gain from one another (Ostrander and Schervish, 1990; Barman, 2017). Charitable behaviour should therefore be understood as relational, rather than a solely individual affair.

In addition to giver-beneficiary relations, connections between givers are also important. For example, in a study on collectivist-based (i.e. group-driven) volunteerism in the US, Eckstein (2001) found that collectivist volunteering is sustained over a long period of time and is deeply embedded in community identities, contrary to the more episodic quality that giving or volunteering takes when framed as individualistic. Similarly, in Malkki’s (2015) work with elderly local Red Cross fundraisers in Finland, volunteers overcame a sense of loneliness and disconnection from wider society, and in Flores’ (2014) examination of charity shop volunteers in the UK, volunteering was framed as relational work in which volunteers formed family-like relationships with one another whilst expressing their care for a particular cause. Fundraising activities have often capitalised on and sought to further facilitate the social nature of giving (Tennant, 2013). As Breeze (2013:16) asserts, “normative assumptions about rational choice do not provide an adequate descriptive model of decision making by charitable donors”. Relatedly, Reich has argued that charity should be researched not merely as “the aggregation of private individual decisions to give money away” (Reich, 2020:26) but as an artefact of the state, deeply structured by laws and regulations. Thus within any given context, charitable behaviour is a socio-relational phenomenon, driven by a mix of individual and structural factors.

### 3.2 The role of the fundraiser: charitable relations as triadic

These approaches usefully highlight the socio-relational, rather than individualistic, nature of charitable behaviour. However, they largely present giving relations as dyadic, focusing on the interactions between givers and beneficiaries. This neglects the role and presence of fundraisers; individual and/or organisational fundraisers who design and run appeals, events, and activities to generate support for a particular cause (Chapman et al, 2022). In so doing, fundraisers construct narratives of intended beneficiaries and convey these to potential donors (Macrae, 2022). This simultaneously raises awareness of a need and solicits support – both of which have been identified as key reasons why people give (Bekkers and Wiepking, 2011). Depending on the cause and context, fundraisers may also decide how funds raised are subsequently spent.

Fundraisers can work on a voluntary basis – in which case they are assumed to be driven by similar motives as other charitable volunteers – or be professional, paid staff. Community fundraising for local causes shrinks the ‘charitable triad’ (Chapman et al., 2022): distinctions between donor, fundraiser, and beneficiary are blurred, with local community members frequently contributing time and/or money, soliciting contributions from others, *and* feasibly benefitting from funds raised as local facilities are enhanced.

Previous research on fundraising in the NHS has yielded significant insights into national appeals (Stewart et al, 2024; Möller, 2025) and broad patterns of fundraising and giving at the regional (i.e. NHS Trust) levels (Abnett et al, 2023; Bowles et al, 2023). However, the perspectives of community fundraisers continue to lack attention – warranting our present study. Responding to calls for research on ‘neglected fundraisers’ (Chapman et al., 2022), we show how community fundraisers in the NHS perceive, construct, and respond to the question of additionality.

## 3 Methods

This paper is based on four case studies (Stake, 1995), selected to represent the regional variation of the NHS charities landscape. We sought to explore a middle range of NHS charities between the very large, often London-based NHS charities which are national, if not global, brands (e.g. the Great Ormond Street Hospital Charity) and the small, essentially dormant organisations which are little more than a legal entity, and which conduct little or no active fundraising (Stewart and Dodworth, 2023).

### 3.1 Outline of Case Studies

Case Study A focuses predominantly on a ‘capital appeal’ to raise money for a scanner for a Scottish health board, which ran between 2018 and 2024. During two periods of fieldwork (in 2019 and 2023-24), Author 2 conducted 14 semi-structured, in-person or telephone interviews with 13 staff and fundraisers and undertook participant observation at four community fundraising events. We also analysed publicly available pages from the appeal website, social media, and news coverage.

Case Study B documents the work of a large NHS charity in an English city. Data was collected by Author 3 between May and October 2023, adopting a “patchwork ethnography” (Günel and Watanabe, 2023) approach, during which the researcher shadowed staff within the organisation and on visits to the local hospitals supported by the charity. Participant observation was conducted during fundraising events and in two charity shops affiliated to this organisation. In total, 21 semi-structured interviews with paid staff and volunteers were conducted, some in person, others remotely via Microsoft Teams or Zoom, depending on participants’ preferences and availability. The organisation’s social media activity was also regularly monitored on X (formerly Twitter) throughout the data gathering period to support findings, better inform questions, and to gain a deeper sense of the reach of the charity’s work and of the public’s engagement with the organisation.

Case Studies C and D involved two focus groups conducted with hospital volunteers, staff, and fundraisers, each at a different hospital, led by Author 1. Both were community hospitals located in and serving rural towns in England. Focus group C took place in December 2023 with seven participants; focus group D took place in January 2024 with 11 participants. Participants were asked about their past and present fundraising activities at their hospital including their motivations for fundraising and/or volunteering, the nature of such activities, how these have changed over time, and their perspectives on how such activity supports, impedes or otherwise intersects with their hospital and the NHS. The websites and social media profiles of both were examined in advance, informing further detailed questions.

Case Study participants are summarised in Table 1. Broadly, these included NHS staff, paid charity staff, unpaid charity committee members (e.g. League of Friends Chair, charity trustee), and volunteers (people who volunteer their time to fundraise for, or otherwise support, the hospital). These categories are deliberately broad, and we do not offer demographic characteristics of participants, to protect the anonymity of participants within case studies. Even assigning people to these categories was challenging, given that participants had often moved between categories, or occupied multiple categories at a time. Where relevant to the point we are making in our findings, we highlight the specific positionality of the participant.

### 3.2 Data analysis

Interview and focus group audio recordings were transcribed by a professional transcription company and later anonymised by researchers. To ensure their anonymity, all participants have been given pseudonyms (a letter signifying which case study they participated in, and a differentiating number, e.g. A1).

Our analytic approach was a reflexive thematic analysis (Braun and Clarke, 2019). Open coding of interview transcripts was done by hand in the first instance by Author 3 and subsequently, in discussion with the team, using the qualitative data analysis software NVivo. A unique set of codes and subcodes was identified in each case-study, some of which surfaced across all four case studies. These included ‘what is being fundraised for’; ‘more than or other than fundraising’; and a series of sub-codes describing fundraising motivations. Once developed, codes were cross-checked again against data. Fieldnotes have been included in our analysis to support the themes identified in each case.

## 4 Findings

Our participants described their choice to take part in fundraising activities in ways which resonate with much of the existing literature on broader charitable volunteering (Mohan, 2024). They depicted fundraising as a rewarding activity, and a highly social one. They described the enjoyment and creativity of coming up with fundraising ideas and they talked about how rewarding it was to have a valued role (particularly if they were retired or otherwise not formally employed). Fun and pleasure featured heavily, unlike in most scholarly analyses of social action. These motivations for taking part in fundraising justified people not only seeking involvement, but in many cases staying involved, devoting significant amounts of time to fundraising for the NHS for years.

We begin this section by considering the material objects which fundraisers were seeking to fund and how these were positioned as essential/statutory or additional/charitable. We then go on to reflect on fundraisers’ perspectives on additionality; reflecting on their understanding of it in principle, and in practice. In so doing, we reflect on the affective response from fundraisers across the cases to the unsettled question of what the state and charity should contribute to healthcare.

### 4.1 Fundraising… for what?

Abnett et al (2023) argue that decades of health policy in the UK has avoided defining the sorts of cause that are acceptably ‘additional’ for NHS fundraising. Their analysis of NHS charity reports in England and Wales between 2014-2019 groups identified funding into items which ‘supplement’ or ‘substitute’ Government provision through the NHS. Their analysis finds the category of supplementing, which is also represented by Marion Allford’s description of a dividing line above, includes spending on research and training, alongside a broad range of patient and staff welfare items, including flowers, art, newspaper and toys to enhance clinical environments. Substitutive spending includes equipment ranging from the routine (pulse oximeters, vital signs monitors) to the advanced (neurosurgical microscopes), and furniture, including mattresses, chairs and cupboards.

In our case studies, fundraisers talked about a wide range of ‘goods’ that their work funded, ranging from community gardening projects and small care packages for patients, to major capital appeals for new hospital buildings or large pieces of equipment. Many examples were of relatively small purchases that fundraising had enabled. This included pieces of medical equipment and furniture which would be considered standard, but for which there was a rationale that it was appropriate charitable spend:

“£72,000 on a brand new operating microscope… Because the Hospital Group, of which we are part of, we always think of ourselves, needed two operating microscopes. They could afford one but they couldn’t afford the second.” (C4, volunteer)

Constructing or assessing this rationale involved how the equipment went beyond the standard care provision, either because of its intrinsic characteristics (such as a new functionality) or because it expanded the quantity of provision above an acceptable, but not optimal, level. Our case studies also included some larger capital appeals. In case study A, fundraising was focused on the larger goal of fundraising to purchase an MRI Scanner, following a previous campaign to buy a CT scanner. These again, are standard pieces of equipment in the NHS, rather than cutting edge new technology being pioneered with charitable funding. However in both cases acquiring a local scanner was considered unaffordable for the local NHS, in terms of priorities for major capital spend, and so charitable fundraising for the scanner enabled patients to avoid lengthy journeys to the next closest alternative equipment.

Patient and staff comforts were often discussed as a target of fundraising. These items allowed fundraisers to draw on their own or loved ones’ experiences of what would have made hospital stays easier. One fundraiser reflected on a project to decorate a neonatal unit with images depicting the positive journeys of previous patients:

“I was telling my mum about it, and she was like “that would have been phenomenal if I’d had that because just to be able to walk along that corridor and be able to see that hope.” (B5, paid charity staff)

However patient comforts were also an opportunity to enhance the experience of people less fortunate than the fundraisers themselves. The same fundraiser gave the example of ‘care packs’ for patients people who were near the end of their lives or did not have a network of social support beyond the hospital, describing them as “extra bits… little extras”.

Charitable purchases for patients were also seen as improving staff experience of their jobs.

“At Christmastime we’ve been able to fund presents and on the old person’s ward, we had some feedback from the matron who was literally welling up when she was saying being able to give that gift to some of her patients, she said she hasn’t seen them have a single visitor and it was potentially their last Christmas. And as a nurse you want to make a difference to your patients. And she said we enabled her to feel good about doing her job. I’m getting quite emotional talking about it now. But, you know, that’s something that’s really simple, you would never expect the NHS to be paying for gifts for patients at Christmas, but you can absolutely recognise the value that it adds to the patient experience and to the member of staff giving that gift.” (B9, paid charity staff)

This last quote, explicitly referencing going beyond what one might “expect” from the NHS, depicts a charitable purchase which benefits not only its direct recipient, but also the staff caring for them. Improvements to the hospital environment, often focused on creating attractive green spaces, were also described as a rewarding goal which benefited patients and staff:

“it’s a beautiful area where people can sit. Staff can go out there and sort of have one-to-one supervision, quiet time, you know, families can take loved ones outside in the nice weather for a walk around the gardens at the hospital as well” (C2, NHS staff)

These quotes point to the sense of collective, and often mutually beneficial, endeavour that characterised the NHS fundraising we studied. Threaded through these accounts was an awareness of questions of additionality. In the next section we explore how fundraisers described navigating this terrain.

### 4.2 Professional fundraiser perspective on additionality

Perhaps unsurprisingly, given that it is a somewhat technical question of charity governance, the dilemma of additionality was more often spontaneously raised by professional staff than it was by voluntary fundraisers. This was particularly evident in Case Study B which was the most professionalised of the four case study contexts. Paid charity staff were usually able to clearly articulate the ‘added value’ role of the charity, and held most consistently to it; “to me that’s what it’s all about, those extras” (B5, paid charity staff). Professional fundraisers (especially those with experience outside NHS charities) also had a clear sense of the distinctive tension between communicating this ‘extra’ or inessential function of their charity, and communicating enough urgency to motivate donors.

“The trust don’t want us to be too critical. So we can’t go out to the public and say this service is terrible, this is all falling down, we need new buildings…you’ll find with NHS charities there’s often a conflict between them and the trusts communication teams. We work very closely with the communication team here. You know, there’s sometimes difficult conversations to be had, they don’t want to as I said to be seen to be offering a diminished service in any way. But we need a burning platform. We need to give the impetus for people to donate. So it’s a tricky balance.” (B1, paid charity staff)

For professional fundraisers used to constructing persuasive messaging, additionality could seem like an obstacle to overcome.

Volunteer fundraisers were, given their less formal roles in charity decision-making, less likely to volunteer dilemmas around additionality in their descriptions of their roles. However, given our research focus, it was a question raised by us in interviews and focus groups with volunteers. The next two sections consider responses from this group.

### 4.3 Volunteeer fundraisers navigating additionality

Across the cases, many volunteer fundraisers held on to the idea that their charitable work contributed nice-to-have but inessential extras to the local NHS, providing examples of what they were and were not willing to fund:

“[we provide] the extras that make the patient experience or the staff experience much better” (C5, volunteer)

Maintaining and clearly articulating this distinction between inessential and essential was sometimes described in terms of actual decision-making, as when particular requests came before charitable committees. Many of the examples of patient amenities given in previous paragraphs, fell into this category. Volunteer fundraisers also presented additionality as particularly important when appealing to donors (at events or fundraising stalls) who, participants believed, increasingly question why fundraising for the NHS takes place at all.

“I think I get asked certainly by so many people ‘why do we have to fundraise for the NHS?’ I mean, it’s a very basic question” (C1, Volunteer)

Maintaining, and communicating, a clear essential-extra binary was presented as an important aspect of fundraising narratives to avoid putting off potential donors.

However, this binary was consistently described as blurred in practice. A particularly revealing exchange took place during the focus group for Case Study D:

“Frankly, any League of Friends is faced with the fact the public perception might be, probably is a lot of time, ‘Well, the NHS should be providing this. Why am I going to pay? I’m already paying my taxes, why should I contribute in addition to pay for something which should be coming by another route?’.[…]” (D10, Volunteer)

“I suppose that’s where we have to clearly define what fundraising we do here is not – it’s extras, it’s not anything to do with the NHS.” (D9, Volunteer)

“Yeah, but there’s still some blurring, isn’t there?” (D10)

“Of course there is, unfortunately.” (D9)

“Like the recliner chairs, which cropped up as a conversation quite a lot, didn’t they? They’re not a clinical need, but nor are they a social need, or nor are they a luxury. They’re part of, most people have an armchair in their sitting room. Should we be – and I’m not saying we shouldn’t be – but should we be paying for those or should they be part of the fabric that come with the basic equipment that’s about patient care?” (D10)

“Realistically they won’t come at all.” (D1, Volunteer)

“Exactly.” (D10)

This exchange illuminates an alternative angle on the dilemma of additionality. While professional fundraisers sought to articulate a “burning platform”, volunteer fundraisers ruminated over how they could justify paying for items that felt too close to “basic equipment that’s about patient care”. The puzzling over what turns a common piece of household furniture into something ‘additional’, often came to rest on functions like reclining, or whether the chair was for the comfort of a patient’s visitors, rather than for the patient themselves.

In addition to debates about ‘blurring’ of the essential-extra binary, in several cases, participants overtly acknowledged that their fundraising was providing what they considered to be basic or essential healthcare.

“every now and again something comes up and says, ‘who’s going to pay for the renewal of the beds?’. Well, now and again we’ll say, ‘we’ll pay for a couple of beds’, only to encourage the fact that we want modern beds there, otherwise they’d sit there for some time. So, we exercise our right to be able to step in and help, if we choose.” (D5, charity committee member)

Similar to the statement above that reclining chairs “won’t come at all”, this quote highlights that old, less functional beds would “sit there for some time” if the charity did not replace them. In Case Study A, where the appeal to buy a scanner was formally an ‘extra’, but widely understood to be necessary, this was especially pronounced:

“It does feel different in some respects. It’s like why are we having to fundraise for this because should they not be supplying it anyway?” (A12, Volunteer)

This volunteer fundraiser explicitly compared her previous experiences raising money for patient comforts in a children’s hospital, to the scanner which in her view the NHS or the Government ‘should’ be providing.

Across the case studies, participants attributed their crossing of the essential-extra binary to the context of an ‘austere’ healthcare system and the post-pandemic period:

“I think the difficulty is that the NHS is obviously, for as long as I can remember, always been struggling. And, of course, the demand is always increasing on the service. And we are now fairly quickly reaching a situation where, let’s say, the general public is almost now going in and funding different things in hospitals. And I think that’s a great shame.” (A6, Volunteer)

“I suppose that the NHS only has a pot of money that’s a certain size and out of that, all of the services have to be provided. And there are more and more people in need of services, as time goes on and the treatments provided for people; there’s cancer treatments and all of these things become more and more expensive and more people are accessing them. So, I suppose that anything that can be done to help, is a good thing” (A5, Volunteer)

“The country here has got an enormous debt. There is no way in which you can just throw money at the NHS forever. So, it is a much better idea, we believe in the [Charity], people who want to throw money at a specific thing, to your hospital, to your Eye Department and things like that, give it there.” (C5, volunteer)

Participants expressed a mix of muted anger and resignation that because the NHS does not have sufficient funds to cover everything, and yet sought to protect local services to which they were deeply committed and attached. In a recent paper, Author 2 has described this orientation as “everyday pragmatism”; “a motivation to act not only despite the circumstances, but in acknowledgement of them” (Vaghi, 2026). Thus, whilst participants expressed both their own preference and a perceived preference amongst donors to only fundraise for ‘extras’, participants in every case study freely gave multiple examples where they felt they had also provided essentials, or where the distinction between essential and extra was at least subjective.

## 5 Discussion

Reich (2020) argues that tradeoffs between liberty and equality are foundational to debates about the role of charity in democracies. Additionality is one route through the dilemma of seeking a system which enables people to devote their time voluntarily to causes important to them, while recognising that this freedom might exacerbate existing inequalities by allocating resources by the appeal or apparent deservingness of the cause, not the scale of the need. Where additionality works, as a principle, it can enable people to be free to devote their energies to their preferred causes, while ensuring that basic needs are met in an equitable fashion through more democratic means.

Previous literature has assumed, based on studies of elite philanthropy, that fundraisers will seek to avoid causes where their contributions might substitute or supplant state funding. This paper has reported the perspectives of community fundraisers and the professional staff working with them from across four case studies of fundraising to support the NHS. Doing so addresses the neglect of community fundraising and fundraisers in past research (Chapman et al, 2022) which is heavily biased towards the perspectives of donors, particularly large-scale donors (Rider, 2019).

Across the case studies examined, participants presented the principal of additionality as a nice aspiration that they, and the donors they engage with, would like to be able to keep to, but one which was often unrealistic in practice. This reflects similar insights from teachers leading fundraising within the education sector (Body, 2020): whilst core costs of publicly-funded services are perceived as the responsibilities of the state, actors nonetheless pursue charitable support where budget are inadequate. Across each of our case studies, participants described the essential-extra binary as inherently blurred and frequently crossed: examples were given in every case study where participants had fundraised for something that could reasonably be considered essential.

Just as donors’ care for a particular cause can override additionality concerns (Breeze, 2010), we found that participants’ overriding concern was not questions of additionality but rather local provision being good enough. Far from being distanced donors, community fundraisers are members of the community they are fundraising for. This proximity affects how decisions are made. In community fundraising, people are not passively being ‘appealed to’ by professional fundraisers but are acting as ‘appealers’ themselves; local settings emerge as the domains where nationwide policy processes can be negotiated more tangibly by people, and specific concerns relevant to different communities can be more directly addressed.

Reflecting the findings of other studies on the motivations for voluntary work, participants described fundraising and volunteering as a social endeavour in which they formed valued relationships. Importantly, this social reward is attained regardless, or even in the absence, of a defined fundraising goal. Supporting Breeze’s (2013) argument about path dependency, this suggests that precisely what community fundraisers are fundraising for – and consequently, potential issues of additionality – becomes less important, so long as it is in some way supporting the local institution they value. Celebrations and enjoyment of fundraising efforts incentivise and normalise further fundraising activities. Fundraising for specific items is, broadly, a ‘fix and mend’ approach (Orton et al, 2011) that both reinforces inequalities in healthcare provision – as communities have different levels of social and financial capital and, therefore, abilities to fundraise (Mohan and Clifford, 2024) – and may distract from upstream efforts aimed at reducing health inequalities.

**Figure 1:**
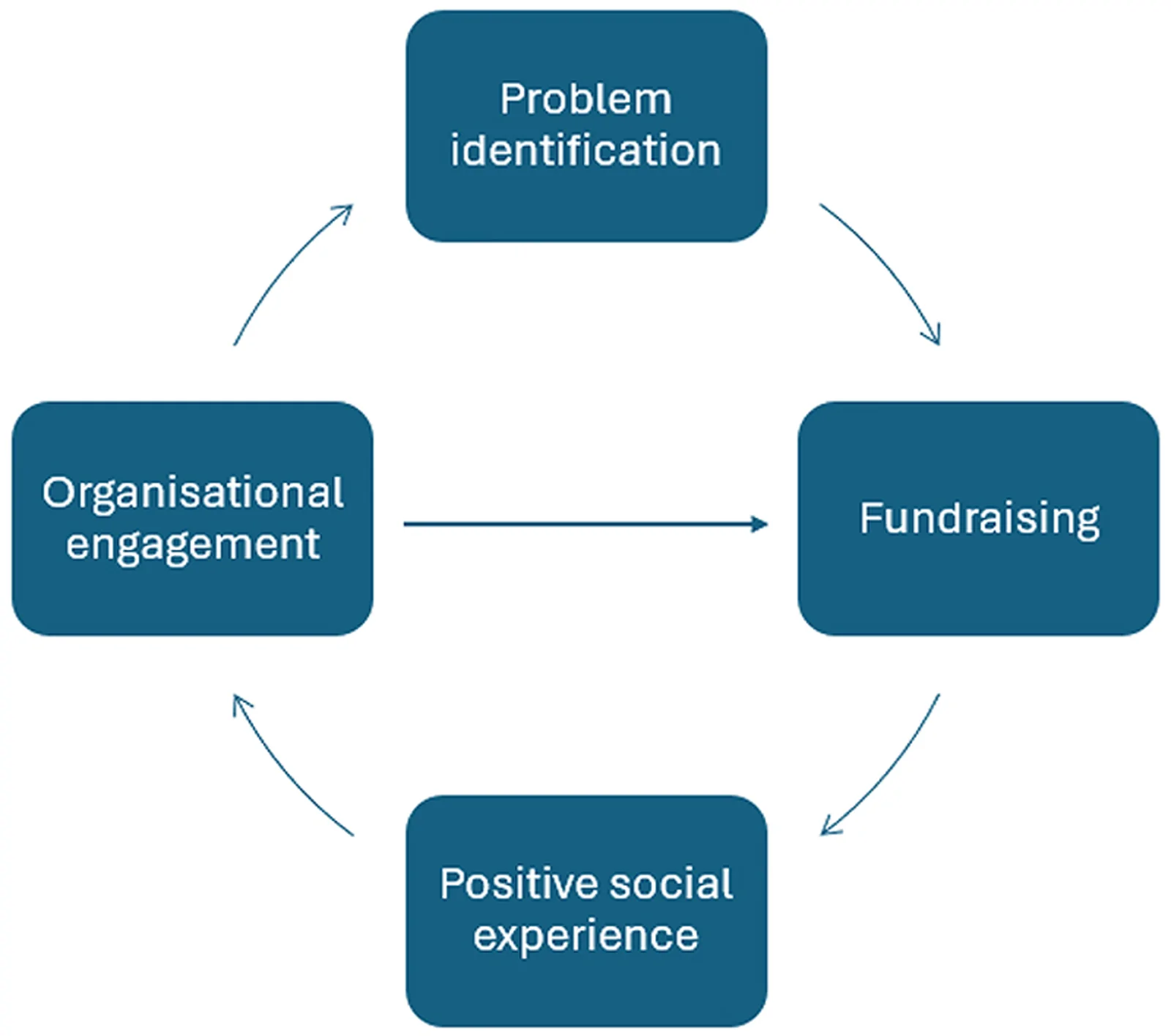
‘virtuous’ cycle of community fundraising for the NHS

In Figure 1, we depict these findings within a ‘virtuous cycle’ of community fundraising, to explain how expressed concerns about the additionality of their contributions might not prevent their ongoing and, in some cases, remarkable commitment to fundraising for local NHS provision. Many of our fundraisers were drawn to the activity by a sense of gratitude for local healthcare and a perception that those services may be at risk or under threat (whether from broader lack of funding, or in some cases from service reconfigurations on clinical grounds); this is the factor labelled ‘problem identification’ in the figure. However, once they had embarked upon a fundraising journey, where this was an enjoyable social experience, they often became committed to an ongoing relationship with the cause in question. What keeps people fundraising and volunteering was not a regular assessment of the appropriateness of a goal (its additionality or otherwise), but the web of friendship, enjoyment and fulfilment that builds up around a charity. Consequently, while the existence of a cohesive fundraising group might also lead to new problem identification (perpetuating the cycle), fundraising activities could also continue in the absence of a defined fundraising goal, as a broader show of support for a valued organisation.

## 6 Conclusions

In theory, charitable contributions for publicly-funded services should be limited to providing inessential, rather than core, resources (Schuster, 1994). However, as our findings highlight, such distinctions remain ill-defined and often blurred. Amidst wider financial pressures on the NHS, professional and volunteer fundraisers described different orientations to navigating this essential-extra binary. For professional fundraisers, used to conveying urgency to attract donations, selling charitable causes as important but not essential was a challenge to overcome. Community fundraisers were just as aware of the tension, but quicker to dismiss additionality as a nice, but frequently unrealistic, aspiration. Their care for local hospitals, and enjoyment of fundraising activities, superseded additionality concerns: community fundraisers are willing to, and often do, provide essential resources to their local hospital through charitable means (Stewart, 2021).

Given cross-community variations in fundraising capacity (Mohan and Clifford, 2024), the path dependency of fundraising threatens equity of health care provision in a national, tax-funded system. Current patterns of NHS community fundraising direct attention to ‘downstream’ solutions which meet immediate needs locally (Stewart, 2026). While NHS charities are being encouraged to focus more on prevention of ill-health (NHS Charities Together, 2023), our findings suggest people who currently fundraise for local hospitals may not be equally motivated by that more diffuse goal. Communities have been framed as ‘renewable energy’ for local healthcare facilities (Paine et al, 2019), but local fundraising reflects patterns of what communities care about and what they have *usually* supported. It cannot simply be redirected externally towards any good cause.

Our findings demonstrate the enduring value of researching fundraising for public goods as a context-dependent and socio-relational phenomenon. They also suggest several avenues for further research. Specifically, while our case studies revealed the perspectives of four groups of long-standing and committed community fundraisers, who were predominantly white British and retired or approaching retirement. Research is needed to explore inter-generational differences in attitudes towards fundraising for publicly-funded services. Research examining younger people’s perspectives on additionality would help shape future conversations and funding decisions for publicly-funded services. Furthermore, as claims about the NHS become co-opted into increasingly populist and nationalistic discourses (Fitzgerald et al, 2020), research should purposively seek to understand how different communities of identity (including ethnic minority communities and migrants) fundraise for, and perceive fundraising for, healthcare in the UK.

## Figures and Tables

**Table 1:** Case study research participant details.

	NHS staff	Paid charity staff	Charity committee members	Volunteers
**Case Study A**	2	2	2	7
**Case Study B**	0	11	1	10
**Case Study C**	4	0	0	3
**Case Study D**	0	0	6	5
**TOTALS**	5	14	9	25

## Data Availability

The dataset underlying this study is deposited in the University of Strathclyde’s PURE repository at https://doi.org/10.15129/b87af8e4-e581-443d-be1f-b511965b8e3c, where participants consented to data archiving. Due to ethical concerns about participant anonymity, not all transcripts reported in this paper are openly available.
